# Analytical and Clinical Evaluation of a Fully Automated Direct-from-Whole-Blood Multiplex PCR Assay Using Large-Volume Reverse Elution for Rapid Bloodstream Infection Diagnosis

**DOI:** 10.3390/antibiotics15090896

**Published:** 2026-09-11

**Authors:** Chi-Sheng Tai, Hsing-Yi Chung, Tai-Han Lin, Chih-Kai Chang, Cherng-Lih Perng, Hung-Sheng Shang, Ming-Jr Jian

**Affiliations:** 1 Graduate Institute of Medical Science, National Defense Medical University, Taipei City 11490, Taiwan; eric7142@gmail.com; 2 Division of Clinical Pathology, Department of Pathology, Tri-Service General Hospital, National Defense Medical University, Taipei City 11490, Taiwan; cindyft12@mail.ndmctsgh.edu.tw (H.-Y.C.); doc31756@mail.ndmctsgh.edu.tw (T.-H.L.); imabacteriaman@mail.ndmctsgh.edu.tw (C.-K.C.); pcl@mail.ndmctsgh.edu.tw (C.-L.P.); 3 Graduate Institute of Pathology and Parasitology, National Defense Medical University, Taipei City 11490, Taiwan

**Keywords:** bloodstream infection, sepsis diagnostics, multiplex PCR, direct-from-blood testing, antimicrobial resistance, ESKAPE pathogens, limit of detection, nucleic acid extraction, antimicrobial stewardship, rapid diagnostics

## Abstract

**Background/Objectives:** Prompt identification of bloodborne pathogens and their antimicrobial resistance is critical for effective sepsis management. Direct molecular testing of blood offers faster results than blood culture; however, its clinical utility has been limited by suboptimal sensitivity and procedural complexity. This study evaluated an automated rapid multiplex PCR assay (BPID^®^ system) and compared its performance with gold-standard methods. **Methods:** This was a preliminary, single-centre diagnostic-agreement study. Forty-three whole-blood samples were collected from patients with suspected bloodstream infection at Tri-Service General Hospital, Taipei, Taiwan, between January 2025 and January 2026, and were analysed. Three molecular methods and two culture-based methods were compared: the BPID^®^ system, a Qiagen^®^-based workflow, the BioFire^®^ FilmArray^®^ BCID2 panel, blood culture, and MALDI-TOF MS identification. Positive percent agreement (PPA) was calculated at the organism level against a composite reference standard. Analytical sensitivity (limit of detection, LoD) was assessed in whole blood spiked with serial dilutions of ten reference organisms. **Results:** The composite reference standard yielded 47 reference-positive organisms across 42 samples. The BPID^®^ system achieved a PPA of 95.74% (95% CI, 85.8–98.8), numerically similar to the post-culture BCID2 panel (93.62%; *p* = 1.00, exact McNemar test), and higher than the Qiagen^®^ workflow (72.34%; *p* = 0.001), blood culture (72.34%; *p* = 0.007), and MALDI-TOF MS (74.47%; *p* = 0.013). In a sensitivity analysis using a composite reference standard from which the index test was excluded, 46 organisms remained reference-positive and the PPA of the BPID^®^ system was 95.65% (44/46; 95% CI, 85.5–98.8), again numerically similar to the BCID2 panel (93.48%; *p* = 1.00). For antimicrobial resistance determinants covered by both panels, the BPID^®^ system reproduced the BCID2 determinant profile in all 16 specimens in which such a determinant was reported (100%), versus 10 of 16 specimens (62.5%) for the Qiagen^®^ method. Confirmed LoD values for the BPID^®^ system were 0.67–20.81 CFU/mL, an 8.8- to 24.3-fold improvement over the Qiagen^®^ method (6.51–224.61 CFU/mL). **Conclusions:** The automated BPID^®^ system substantially improves PCR-based diagnostic sensitivity for bacteremia and, by operating directly on whole blood, removes the culture-incubation step that post-culture molecular panels require. Because the study was not designed as an equivalence or non-inferiority trial and evaluated only 43 specimens, the similar agreement observed for the BPID^®^ system and the BCID2 panel should not be interpreted as demonstrated equivalence; these findings should be regarded as a preliminary clinical evaluation requiring confirmation in larger multicentre studies that also enrol culture-negative and uninfected controls.

## 1. Introduction

Bloodstream infections represent a leading cause of morbidity and mortality in hospitalized patients worldwide, with sepsis-related fatalities imposing a significant burden on healthcare systems [1,2]. Timely identification of causative pathogens and their antimicrobial resistance profiles is essential for guiding targeted antimicrobial therapy, reducing inappropriate broad-spectrum antibiotic use, and improving patient outcomes [3]. Current gold-standard diagnostic workflows rely on blood culture followed by organism identification using matrix-assisted laser desorption/ionization time-of-flight mass spectrometry (MALDI-TOF MS) and phenotypic antimicrobial susceptibility testing, a process that typically requires 48–72 h from sample collection to actionable results [4,5].

The emergence and spread of multidrug-resistant organisms, particularly the ESKAPE pathogens (*Enterococcus faecium*, *Staphylococcus aureus*, *Klebsiella pneumoniae*, *Acinetobacter baumannii*, *Pseudomonas aeruginosa*, and *Enterobacter* spp.), has intensified the urgency for rapid antimicrobial resistance detection [6,7]. Delayed recognition of resistance determinants such as *mecA/C*, CTX-M, NDM, KPC, and OXA-48-like genes can lead to prolonged empirical therapy, increased selective pressure for resistance, and adverse clinical outcomes [8,9].

Nucleic acid amplification tests, including syndromic multiplex PCR panels such as the BioFire^®^ FilmArray BCID2, have demonstrated improved pathogen detection compared with culture alone [10,11]. However, most commercially available nucleic acid amplification test-based panels require prior blood culture enrichment, thereby retaining the inherent time-to-positivity delay of culture-dependent workflows. Direct-from-blood molecular testing represents a promising alternative that could bypass the culture step entirely, but existing approaches have been limited by several technical challenges: (1) the low concentration of circulating pathogen DNA in bacteremic blood (often <10 CFU/mL); (2) the dilution effect inherent in scaling up nucleic acid extraction volumes; and (3) the co-extraction of PCR inhibitors from large-volume blood specimens [12,13].

**Figure 1:**
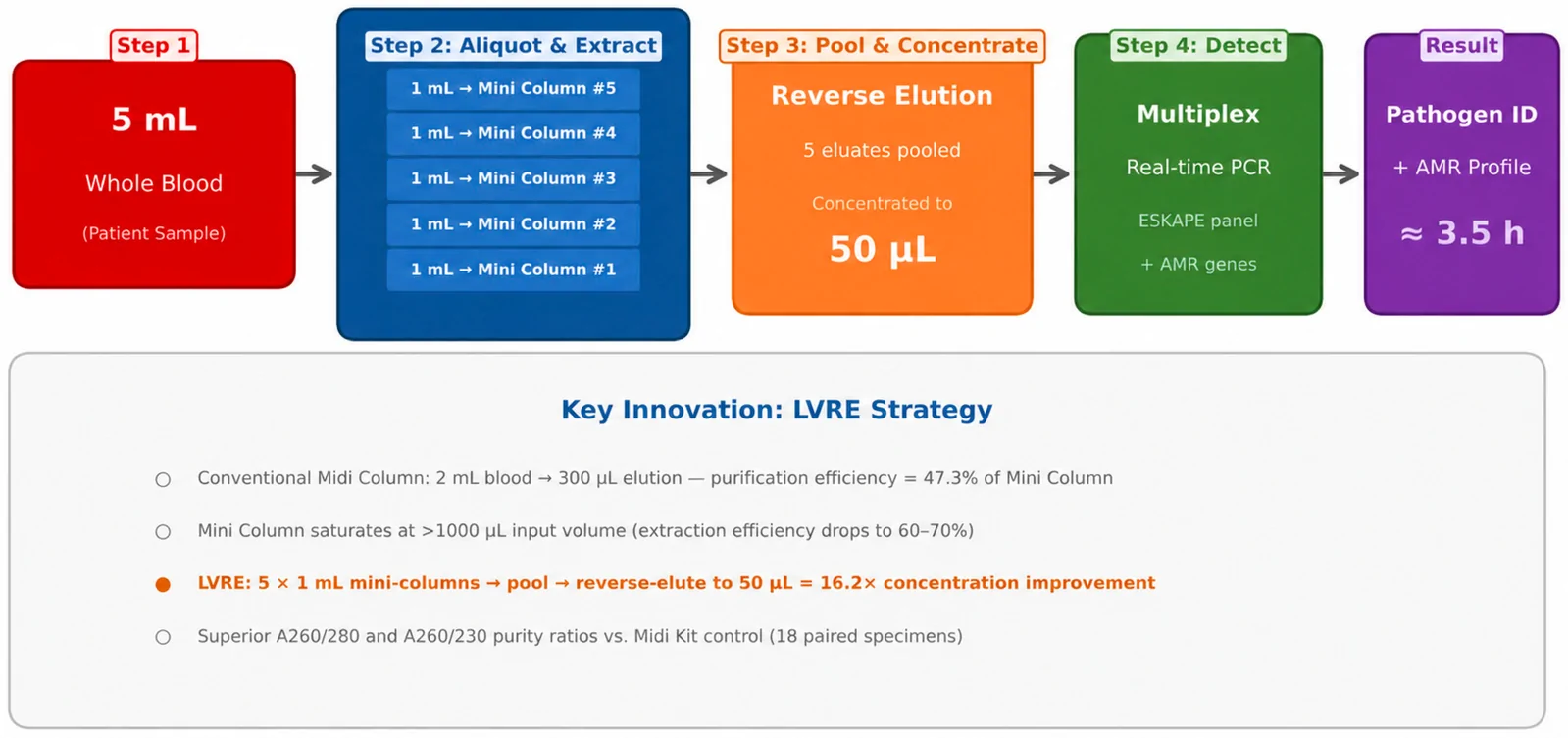
Schematic of the large-volume reverse-elution (LVRE) nucleic acid extraction strategy. Whole blood (5 mL) is divided into five 1 mL aliquots, each processed through an independent silica mini-column; the five eluates are then pooled and concentrated into a single 50 µL eluate by reverse elution, which is used directly for real-time multiplex PCR detection of pathogen identification targets and antimicrobial resistance genes. The lower panel summarizes the rationale for the strategy: a conventional Midi column processing 2 mL of blood with a 300 µL elution achieves 47.3% of the purification efficiency of a mini column, and mini-column extraction efficiency falls to 60–70% at input volumes above 1000 µL. Across 18 paired specimens the LVRE strategy achieved improvement in nucleic acid concentration with superior A260/280 and A260/230 purity ratios relative to the Midi kit control.

To address these challenges, we developed a large-volume reverse-elution (LVRE) nucleic acid extraction strategy that processes five 1 mL aliquots of whole blood through five independent mini-column extraction units, concentrating the recovered nucleic acids into a 50 μL eluate via a reverse-elution technique. This approach yielded higher nucleic acid concentrations compared with conventional Qiagen^®^ Midi Column extraction from 2 mL blood, while maintaining superior purity ratios (A260/280 and A260/230) (Figure 1; Supplementary Figure S1) [14]. The LVRE strategy has been integrated into the LabTurbo^®^ AIO BPID system, a fully automated platform that performs nucleic acid extraction, PCR reaction setup, and real-time multiplex PCR detection within a single instrument.

This study aimed to evaluate the analytical sensitivity (limit of detection [LoD]) and clinical diagnostic performance of the BPID^®^ system by comparing it with the BioFire^®^ BCID2 panel (post-culture molecular reference), a conventional Qiagen^®^-based direct-from-blood workflow, standard blood culture, and MALDI-TOF MS identification. Because the clinical component was exploratory in size and was not powered for a formal equivalence or non-inferiority comparison, it is reported here as a preliminary diagnostic-agreement evaluation.

## 2. Results

### 2.1. Analytical Sensitivity (Limit of Detection, LoD)

#### 2.1.1. Possible LoD Determination

Serial dilutions of ten reference organisms representing the ESKAPE group and additional clinically relevant pathogens were tested in triplicate to establish the preliminary limit of detection (LoD) for both extraction methods, namely the QIAamp DNA Blood Midi Kit (Qiagen, Hilden, Germany) and the LabTurbo AIO BPID Mini Kit (LabTurbo Biotech, Taipei, Taiwan). The concentration at which all three replicates yielded positive detection (100% detection rate) was identified as the possible LoD (Table 1).

Across all ten target organisms, the BPID Mini Kit extraction demonstrated a consistent 10-fold improvement in possible LoD compared with the QIAamp Midi Kit, attributable to the LVRE strategy’s increased blood input volume (5 mL vs. 2 mL) and concentrated elution.

#### 2.1.2. LoD Confirmation

LoD confirmation was performed using 20 replicates at four serial concentrations near the possible LoD. The confirmed LoD was defined as the lowest concentration achieving ≥95% detection rate (Table 2).

**Figure 2:**
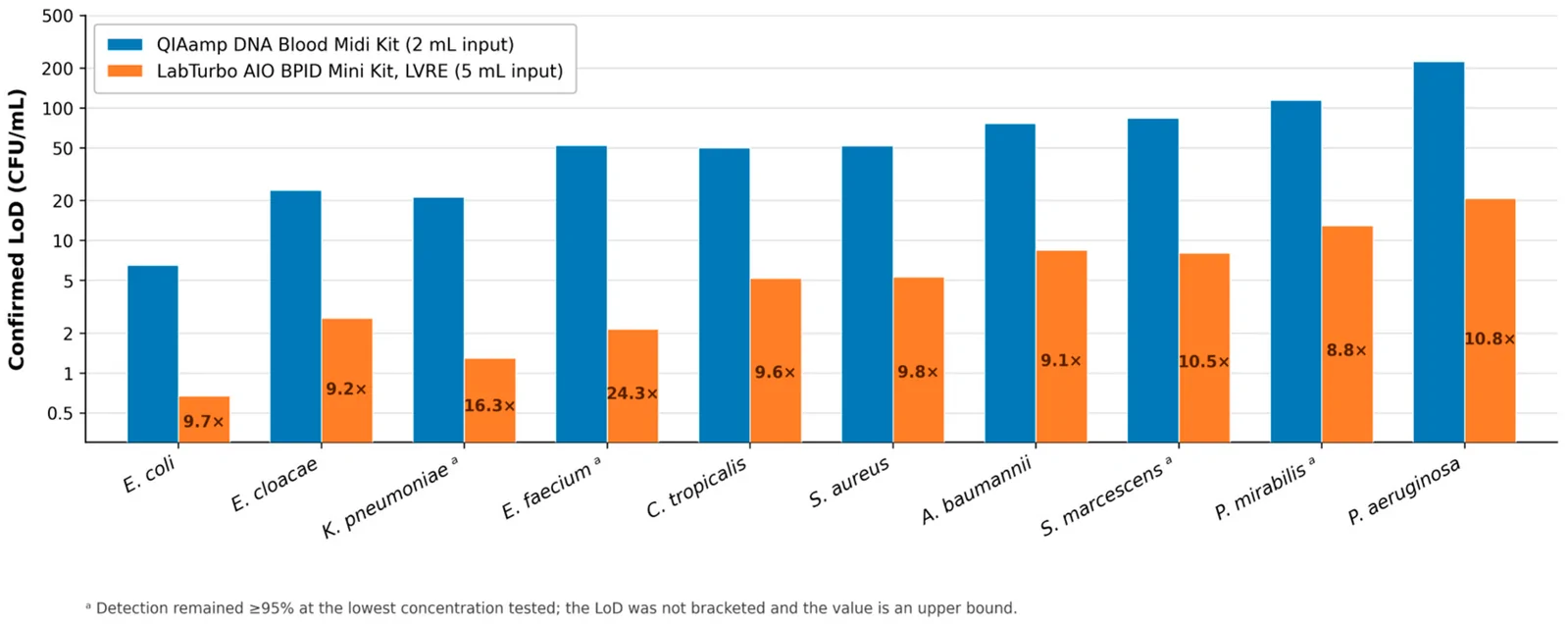
Confirmed limit of detection (LoD) for the QIAamp DNA Blood Midi Kit (2 mL input) and the LabTurbo AIO BPID Mini Kit using the LVRE strategy (5 mL input), for ten reference organisms. The confirmed LoD is the lowest concentration at which at least 95% of 20 replicates were detected. Bars are plotted on a logarithmic scale; the value above each orange bar is the fold improvement relative to the Midi kit for that organism. Improvement ranged from 8.8-fold (*Proteus mirabilis*) to 24.3-fold (*Enterococcus faecium*). For *E. faecium*, *Klebsiella pneumoniae*, *Serratia marcescens* and *P. mirabilis*, detection remained at or above 95% at the lowest concentration tested, so those values are upper bounds. Underlying replicate Ct data are given in Supplementary Tables S1 and S2.

The BPID Mini Kit (LVRE extraction) achieved confirmed LoD values ranging from 0.67 to 20.81 CFU/mL (colony forming units/mL) across the ten target organisms, compared with 6.51 to 224.61 CFU/mL for the QIAamp Midi Kit extraction (Figure 2). The improvement ranged from 8.8-fold to 24.3-fold. For four organisms (*E. faecium*, *K. pneumoniae*, *S. marcescens*, and *P. mirabilis*), detection remained ≥95% at the lowest concentration tested, so the stated values represent upper bounds rather than bracketed LoDs. The coefficient of variation for Ct values at the confirmed LoD concentrations ranged from 1.03% to 3.38% across all organisms for the BPID Mini Kit, indicating acceptable reproducibility. Extraction control Ct values remained stable across all runs (mean range: 22.0–25.3), confirming consistent extraction performance.

### 2.2. Clinical Evaluation

#### 2.2.1. Pathogen Identification

Forty-three clinical whole blood samples were analyzed across five diagnostic methods. A composite reference standard was applied at the organism level, classifying an organism as reference-positive when identified by at least two of the five methods. This yielded 47 reference-positive organisms across 42 samples; one sample (Sample 20, *Pseudomonas oryzihabitans* recovered by culture alone) and two additional single-method identifications (the organisms reported by MALDI-TOF MS alone in Sample 2) did not meet the composite criterion and were excluded from the analysis (Table 3; per-case adjudication in Supplementary Table S4, and the complete listing of discordant results in Supplementary Table S5).

The BPID^®^ system demonstrated the highest PPA (95.74%; 95% CI 85.8–98.8) among all methods tested, and the difference from the post-culture BCID2 panel (93.62%) did not reach statistical significance (*p* = 1.00, exact McNemar test). Because the study was not designed as an equivalence or non-inferiority trial and the number of discordant pairs was very small, this result indicates only that no difference was detected, not that the two assays are equivalent; the BPID^®^ system, however, achieved this level of agreement without any culture enrichment step. Both false negatives arose from a single sample (Sample 24) containing *Acinetobacter schindleri* and *Acinetobacter lwoffii*, organisms not included in the BPID detection panel. The BPID^®^ system outperformed the Qiagen^®^ workflow (72.34%; *p* = 0.001), blood culture (72.34%; *p* = 0.007), and MALDI-TOF MS (74.47%; *p* = 0.013). The Qiagen^®^ method’s 13 false-negative results were attributable to failure to detect the organism, most frequently *Escherichia coli* and coagulase-negative staphylococci.

#### 2.2.2. Antimicrobial Resistance Gene Detection

Among the 43 clinical samples, 16 contained organisms harbouring antimicrobial resistance genes. The LabTurbo AIO BPID Testing Kit (LabTurbo Biotech, Taipei, Taiwan) reports *mecA/C*, CTX-M, KPC, NDM, OXA-48-like and *vanA/B*; the Qiagen-based direct-from-blood workflow uses the identical detection kit and therefore covers the same six determinants, differing only in the nucleic acid extraction step. The BioFire FilmArray BCID2 panel (BioFire Diagnostics/bioMérieux, Salt Lake City, UT, USA), used here as the post-culture molecular reference, reports *mecA/C, mecA/C* and MREJ, *vanA/B*, CTX-M, IMP, KPC, NDM, OXA-48-like and VIM. Only determinants covered by both the index assay and the reference panel were included in the concordance analysis, which was performed at the specimen level: a specimen was counted as concordant when the method reported the same set of shared-coverage determinants as the BCID2 reference (Table 4).

The BPID^®^ system reproduced the BCID2 determinant profile in all 16 specimens, with no discordant specimen. Six specimens were discordant for the Qiagen^®^ workflow, in every case because the organism carrying the determinant was not detected at all rather than because determinant amplification failed. Because several determinants were represented by only one or two specimens, no determinant-specific agreement estimate is reported.

#### 2.2.3. Sensitivity Analysis Using a Reference Standard That Excludes the Index Test

Because the primary composite reference standard incorporated the index test, a post hoc sensitivity analysis, performed at the request of a reviewer, was carried out in which an organism was classified as reference-positive only when it was identified by at least two of the four non-index methods (blood culture, MALDI-TOF MS, FilmArray BCID2 and the Qiagen-based workflow). Under this definition 46 of the 47 organisms remained reference-positive, distributed across 42 specimens. One organism lost reference-positive status because FilmArray BCID2 was the only non-index method that reported it: *Staphylococcus epidermidis* in Sample 41. The PPA of the BPID^®^ system under the index-excluded standard was 95.65% (44/46; 95% CI, 85.5–98.8), compared with 93.48% (43/46) for the BCID2 panel (*p* = 1.00, exact McNemar test), 73.91% (34/46) for both blood culture (*p* = 0.013) and the Qiagen^®^ workflow (*p* = 0.002), and 76.09% (35/46) for MALDI-TOF MS (*p* = 0.022). The point estimate for the BPID^®^ system fell by only 0.09 percentage points relative to the primary analysis (95.74% to 95.65%), because almost every organism detected by the BPID^®^ system was independently corroborated by at least two non-index methods; the two false negatives (the off-panel *Acinetobacter* species in Sample 24) were unaffected. The rank order of the five methods and the direction of every pairwise comparison were unchanged. The complete analysis is given in Supplementary Table S6, and all discordant organism-level results are listed individually in Supplementary Table S5.

### 2.3. Blood Culture Control Experiments

Sixteen blood culture bottles were processed as controls: ten controls of non-detection and six off-panel specificity controls (Table 5). These are designated Group B and Group C; a further group of bottles inoculated with on-panel control organisms (Group A) was not prepared, for the reasons set out in Section 4.7. None of the ten Group B bottles flagged positive during the full five-day incubation period, and neither culture nor the BPID^®^ system returned any detection, indicating that the workflow does not generate a signal in the absence of target nucleic acid. All six Group C bottles flagged positive, with times to positivity between 13.2 and 18.6 h, and each organism was correctly identified to species level by MALDI-TOF MS with a score of ≥2.0, confirming that the intended organism was present and viable in the bottle at the inoculum used. None of the six was reported by the BPID^®^ system, and no cross-reactive on-panel identification occurred; in particular, *Enterococcus gallinarum* ATCC 49573 produced no *vanA/B* signal despite carrying the intrinsic *vanC-1* determinant. Because each organism was demonstrably present, viable and correctly identifiable in its bottle, the absence of a BPID^®^ report reflects the intended boundary of the detection panel rather than a failure of the specimen or of organism recovery.

## 3. Discussion

This study demonstrates that the BPID^®^ system, employing a novel LVRE nucleic acid extraction strategy, achieves substantially improved analytical sensitivity for direct-from-blood multiplex PCR detection of bloodstream infection pathogens, together with higher organism-level agreement than the designated study blood culture set, MALDI-TOF MS and a conventional molecular extraction workflow.

The central technical innovation underlying the BPID^®^ system’s performance advantage is the LVRE extraction strategy. Conventional approaches to increasing blood input volume for nucleic acid extraction face a fundamental trade-off; larger silica membrane columns (e.g., Midi or Maxi columns) require proportionally larger elution volumes, thereby diluting the recovered nucleic acids and negating the benefit of processing more blood. Our experimental data demonstrated that the Midi column achieves only 47.3% of the purification efficiency of the Mini column and that Mini column extraction efficiency declines to 60–70% at input volumes exceeding 1000 μL. The LVRE strategy circumvents this limitation by processing each 1 mL aliquot of whole blood through a separate Mini column, then concentrating the pooled eluates from 5 mL of blood into 50 μL via reverse elution. This approach achieved a mean 16.2-fold increase in nucleic acid concentration across 18 paired specimens, with superior A260/280 and A260/230 purity ratios compared with the Qiagen^®^ Midi Kit control, indicating effective removal of PCR inhibitors (Supplementary Figure S1).

The confirmed LoD values for the BPID Mini Kit (0.67–20.81 CFU/mL) represent an 8.8- to 24.3-fold improvement over the QIAamp Midi Kit (6.51–224.61 CFU/mL). For four organisms (*E. faecium*, *K. pneumoniae*, *S. marcescens*, and *P. mirabilis*), detection remained at or above 95% at the lowest concentration tested, so the corresponding values represent upper bounds rather than bracketed detection limits. Notably, the BPID system achieved a sub-CFU/mL detection limit for *E. coli* (0.67 CFU/mL), approaching the theoretical sensitivity limit of culture-based methods, and confirmed LoD values below 10 CFU/mL for 8 of the 10 target organisms. These values are clinically relevant, as bacteremia in adult patients is frequently characterized by pathogen concentrations below 10 CFU/mL [15,16], a range in which conventional molecular extraction methods demonstrate inconsistent detection.

**Figure 3:**
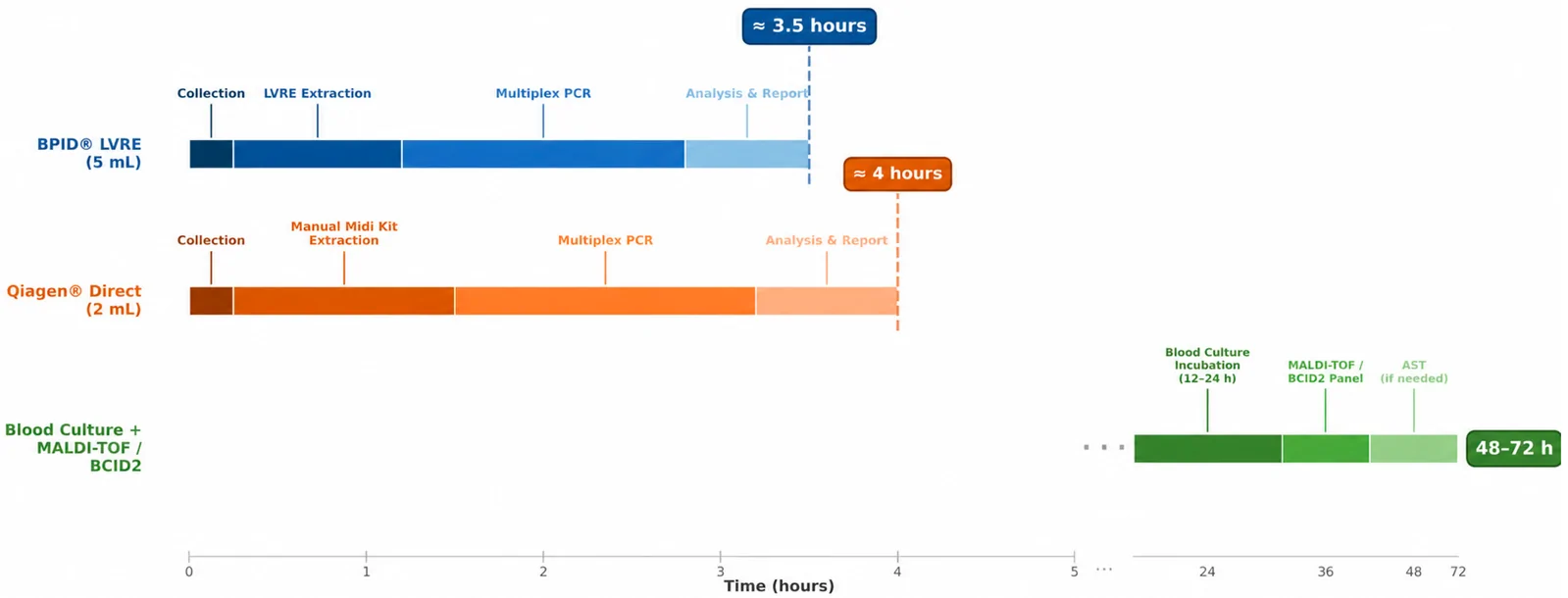
Comparison of diagnostic workflow timelines. The intervals shown are the durations specified by each workflow’s protocol together with the standard incubation and processing times of the culture-dependent pathway; they are not measured turnaround times in the clinical cohort. The BPID system with LVRE extraction (5 mL whole blood) reports pathogen identification and resistance gene results in approximately 3.5 h from sample loading, and the Qiagen direct-from-blood workflow (2 mL, manual extraction) in approximately 4 h. Both bypass culture entirely. The culture-dependent pathway requires 12–24 h of blood culture incubation before MALDI-TOF MS identification or the BioFire FilmArray BCID2 panel can be performed, and 48–72 h in total when phenotypic antimicrobial susceptibility testing is required. Note the discontinuous time axis beyond 5 h.

From an antimicrobial stewardship perspective, the clinical implications of the BPID^®^ system’s performance are substantial. The sample-to-result turnaround time specified by the manufacturer’s protocol for the automated BPID^®^ workflow is approximately 3.5 h, which compares favourably with the 12 to 24 h of blood culture incubation required before a post-culture molecular panel such as the BioFire^®^ FilmArray^®^ BCID2 can be performed, and with the 48 to 72 h needed for conventional culture followed by phenotypic susceptibility testing (Figure 3). Each hour of delay in administering appropriate antimicrobial therapy in septic patients has been associated with measurably increased mortality risk [17,18]. Identification of resistance determinants such as *mecA/C* for MRSA discrimination, NDM and KPC for carbapenem resistance, and CTX-M for extended-spectrum β-lactamase production within a single working shift of specimen receipt would enable clinicians to de-escalate empirical broad-spectrum regimens or to escalate to targeted agents considerably earlier than current workflows permit. In an era of rising antimicrobial resistance, such timely resistance profiling directly supports antimicrobial stewardship goals by reducing unnecessary broad-spectrum antibiotic exposure and its associated selective pressure [19,20]. The comparison in Figure 3 is between the protocol-specified duration of each workflow and the incubation and processing intervals that the culture-dependent pathway requires; it is not a comparison of measured turnaround times in this cohort, which were not recorded. In practical terms, a result available within one working shift allows the antimicrobial stewardship team to act on the same day the specimen is drawn rather than on the following day: detection of *mecA/C* in a staphylococcal infection supports continuation of an anti-MRSA agent, whereas its absence supports early switching to a β-lactam, which is associated with better outcomes in methicillin-susceptible *Staphylococcus aureus* bacteraemia; the absence of carbapenemase and extended-spectrum β-lactamase determinants in an *Enterobacterales* infection supports de-escalation from empirical carbapenem therapy, while their detection supports early escalation and the initiation of contact precautions. Realising this benefit requires a defined notification pathway, because a molecular result only changes management if it reaches the prescriber and is acted upon. Two caveats must be attached to any such pathway. First, the assay reports genotype rather than phenotype and does not replace phenotypic susceptibility testing, which remains necessary for definitive therapy and for organisms with resistance mechanisms outside the panel. Second, a negative result does not exclude bloodstream infection, since the panel is targeted and negative percent agreement was not established in the present study; blood cultures must therefore continue to be collected in parallel. The stewardship benefits described here are mechanistically plausible but were not measured in this study, and prospective evaluation of time to appropriate therapy, days of broad-spectrum therapy and de-escalation rates is required before clinical benefit can be claimed.

In the clinical evaluation, the conventional culture-based methods demonstrated lower pathogen detection rates than the molecular platforms, largely attributable to the nine samples in which the designated study blood culture set showed no growth. Blood culture and MALDI-TOF MS produced organism-level PPA values of 72.34% and 74.47% respectively, and the organisms each method missed were not the same. These culture-dependent methods are inherently limited by their reliance on organism viability and on the organism’s ability to proliferate under standard conditions, which may result in false-negative outcomes when pathogens are present at low concentrations, are in a non-culturable state, or have been partially suppressed by prior antibiotic exposure [21]. Culture-based methods also demonstrated limitations in detecting polymicrobial infections; blood culture recovered only one of the reference-positive organisms in each of three samples (Samples 18, 32, and 41), and MALDI-TOF MS returned no identification in nine samples. The discrepancy between recovery of an organism in culture and failure of MALDI-TOF MS to identify it deserves specific comment, because it is the reason the two culture-dependent methods do not fail on the same organisms despite reaching closely similar agreement values. Four mechanisms were operative in this cohort. First, in polymicrobial specimens the subculture plate yielded mixed growth, and spectra acquired from mixed or insufficiently isolated colonies do not reach the score threshold required for reliable species assignment. Second, organisms recovered only after prolonged incubation, or growing as sparse colonies, provided insufficient biomass for a confident spectrum at the time the specimen was processed within the study window. Third, some isolates yielded identification scores below the cut-off applied in our accredited workflow (≥2.0 for species-level assignment), and were therefore reported as no reliable identification rather than being force-assigned. Fourth, species that are under-represented in the reference spectral library, including the *Acinetobacter* species recovered from Sample 24 and several coagulase-negative staphylococci, cannot be resolved to species level by this method. These are recognised limitations of MALDI-TOF MS applied to primary clinical isolates rather than analytical failures specific to this study, and they illustrate why an identification method that operates on nucleic acid rather than on cultivable biomass can recover information that the culture-dependent pathway loses.

The molecular platforms demonstrated a clear advantage in polymicrobial detection. Both the BPID^®^ system and the BCID2 panel identified all reference-positive organisms in the four polymicrobial samples (Samples 18, 24 excepted for off-panel organisms, 32, and 41), since multiplex PCR can simultaneously detect multiple pathogen targets with high specificity, independent of the competitive growth effects that occur during culture.

The lower clinical performance of the Qiagen^®^ method (organism-level PPA 72.34%) was driven by false-negative results arising from failure to detect organisms, most frequently *E. coli* and coagulase-negative staphylococci. This reduced sensitivity is likely attributable to the smaller blood input volume (2 mL versus 5 mL) and to the dilution effect of the larger elution volume inherent in Midi Column extraction, consistent with the analytical data demonstrating an 8.8- to 24.3-fold difference in confirmed LoD relative to the BPID system. Because both workflows used the same multiplex PCR detection kit and differed only in the extraction step, this comparison isolates the contribution of the LVRE strategy itself.

A methodological caveat applies to all of the agreement estimates reported here. In the primary analysis an organism was classified as reference-positive when it was identified by at least two of five methods, and the BPID^®^ system was one of those five. The index test therefore contributed to the definition of the reference standard, a design that is known to inflate the apparent sensitivity of the index test—an effect usually described as incorporation bias. The bias operates in a specific way: an organism detected by the BPID^®^ system and by exactly one other method becomes reference-positive and is scored as a true positive for the BPID^®^ system, whereas the same organism detected by only one non-index method would have been excluded from the denominator altogether. The reported PPA of 95.74% should therefore be read as an upper bound rather than as an unbiased estimate of sensitivity. The sensitivity analysis presented in Section 2.2.3, in which the reference standard was constructed from the four non-index methods only, was performed to quantify this effect. Reconstructing the reference standard from the four non-index methods removed one of the 47 organisms from the denominator and reduced the PPA of the BPID^®^ system from 95.74% to 95.65% (44/46), a difference of 0.09 percentage points; the ranking of the five methods and the direction of every pairwise comparison were unchanged. The bias introduced by incorporation was therefore small in this dataset, because the great majority of BPID^®^ detections were corroborated by two or more non-index methods rather than by a single one. This should not be read as showing that incorporation bias is unimportant in general; it shows only that in a cohort with a high proportion of culture-positive, unambiguous infections there was little room for the index test to create its own reference-positive calls. The residual comparison against the Qiagen^®^ workflow is also not fully independent, because the two workflows share the same multiplex PCR detection chemistry and differ only in extraction, so that comparison isolates the contribution of the extraction strategy but cannot be regarded as a comparison of two independent assays. A definitive estimate of sensitivity and specificity would require an externally adjudicated clinical reference standard applied by assessors blinded to the index-test result, which was beyond the scope of this preliminary evaluation.

It is equally important to be clear about what the comparison with the BCID2 panel does and does not show. The BCID2 panel was applied to blood culture broth after the bottle had flagged positive, whereas the BPID^®^ system was applied directly to whole blood at the time of the original draw. The two assays therefore occupy different positions in the diagnostic pathway and are not interchangeable comparators: BCID2 is conditional on a growth-positive culture and is, by construction, applied to a specimen containing a high organism burden, while the BPID^®^ system must detect organisms at the concentrations actually present in circulating blood. The similar organism-level agreement of the two assays should accordingly be interpreted as showing that direct testing of whole blood can identify the same organisms that a post-culture panel identifies, without the preceding 12 to 24 h of incubation. The principal potential advantage of the BPID^®^ system is thus the removal of the culture incubation step from the diagnostic pathway, not superior analytical or clinical accuracy relative to a post-culture panel.

These findings can be placed alongside earlier culture-independent approaches to bloodstream infection. The best-studied first-generation direct-from-blood PCR assay, LightCycler SeptiFast, achieved a pooled sensitivity of approximately 68% against blood culture in a systematic review, a level of performance widely attributed to the small blood input volume (1.5 mL) and to inhibitor carry-over [12]. The T2Bacteria panel, the first direct-from-blood assay cleared by the United States Food and Drug Administration, reported a per-patient sensitivity of 90% and a specificity of 90% for proven bloodstream infection caused by its five target organisms in a multicentre study of 1427 patients, with a mean time to species identification of 3.6 to 7.7 h, but it does not report antimicrobial resistance determinants [22]. Plasma microbial cell-free DNA sequencing offers far broader organism coverage but currently requires a centralised laboratory and reports on the day after specimen receipt, and its clinical specificity remains difficult to define because non-viable and colonising organisms are also detected [23]. Against this background, the distinguishing feature of the present work is not the multiplex PCR chemistry, which is conventional, but the extraction strategy: by processing 5 mL of whole blood through five parallel mini columns and concentrating the eluates into 50 µL, the LVRE approach addresses the volume–concentration trade-off that has limited earlier direct-from-blood assays, and it does so within a fully automated instrument that also performs PCR setup and detection and simultaneously reports six resistance determinants. The confirmed LoD values obtained here (0.67–20.81 CFU/mL) are in the range required to detect the low-level bacteraemia typical of adult patients. Whether this analytical advantage translates into a clinical advantage over the assays cited above cannot be determined from a 43-specimen preliminary evaluation and requires a head-to-head prospective study.

Despite these advantages, targeted molecular assays remain limited by the organisms included in their detection panels. In this study, one sample (Sample 24) contained *A. schindleri* and *A. lwoffii*, neither of which is included in any of the molecular panels evaluated; these two organisms accounted for both false-negative results recorded for the BPID^®^ system and highlight the inherent limitation of panel-based molecular diagnostics.

Several limitations should be acknowledged, and together they define this work as a preliminary clinical evaluation rather than a definitive assessment of diagnostic accuracy. First, the clinical evaluation was based on a small sample size (43 specimens yielding 47 reference-positive organisms) from a single tertiary medical centre. With so few observations, the confidence intervals around the organism-level agreement estimates are wide, and the estimates for individual organisms and for individual resistance determinants—several of which were represented by only one or two isolates—are not robust and should be regarded as descriptive only. Larger multicentre studies are required before organism-specific performance can be stated with confidence. Second, only specimens from patients with suspected bloodstream infection were analysed, and culture-negative and uninfected control specimens were not enrolled. Consequently only positive percent agreement could be assessed; negative percent agreement, clinical specificity and the false-positive rate of the assay in patients without bloodstream infection remain unknown, and no estimate of positive or negative predictive value can be derived from these data. Extending the evaluation to a consecutive cohort that includes culture-negative specimens is the single most important next step, and is planned as part of the multicentre study now in preparation. Third, the composite reference standard incorporated the index test, and two of the five comparators (the BPID^®^ system and the Qiagen^®^ workflow) shared the same multiplex PCR detection chemistry and were therefore not fully independent; both features may bias agreement estimates upward. In the opposite direction, the blood culture column of Supplementary Table S3 records the result of the study set only; where more than one set was drawn, an organism may have been recovered from a concurrently drawn clinical set. The agreement estimate reported for blood culture therefore describes the performance of a single study set rather than that of blood culture as a clinical service, and understates the latter. Fourth, the study was designed as a diagnostic-agreement study and was not powered as an equivalence or non-inferiority trial; no equivalence margin was specified in advance. The absence of a statistically significant difference between the BPID^®^ system and the BCID2 panel therefore reflects the limited number of discordant pairs available for the McNemar test and must not be interpreted as evidence that the two assays perform equivalently. In addition, the two assays are applied at different points in the diagnostic pathway, as discussed above. Fifth, the BPID panel, like all targeted molecular assays, cannot detect off-panel organisms; metagenomic next-generation sequencing may offer a complementary unbiased approach. Sixth, the clinical impact of the BPID system on patient outcomes, including time to appropriate therapy, length of intensive care unit stay, and mortality, was not assessed and represents an important avenue for future prospective trials. Finally, the cost-effectiveness of the BPID system relative to existing workflows was not formally evaluated, although reduced hospital stays, decreased broad-spectrum antibiotic use, and improved stewardship may offset assay cost. A further consideration is that the instrument and the test kits used for the index assay were supplied by their manufacturer, as stated in the Funding section, although all testing, analysis and reporting were carried out by the investigators. A further limitation concerns specimen volume. The LVRE workflow requires 5 mL of whole blood, distributed as five 1 mL aliquots. This volume is readily obtained in adults but is not feasible in neonates and small infants, in whom recommended volumes for a complete blood culture set are of the order of 1–1.5 mL and in whom the total volume drawn for diagnostic purposes should not exceed roughly 1% of the circulating blood volume [24]. This is a particularly unfortunate constraint, because low-level bacteraemia at concentrations below 10 CFU/mL is more common in neonates and young children than in adults, and it is precisely this population in which a highly sensitive culture-independent assay would be most valuable [25]. The present data cannot be extrapolated to paediatric or neonatal patients, and a reduced-volume configuration of the LVRE workflow—for example, fewer parallel columns with a correspondingly smaller elution volume—would need to be developed and validated separately, with its own LoD determination, before the system could be considered for use in this population. The control experiments performed for this revision comprised uninoculated bottles and off-panel specificity controls but not bottles inoculated with characterised on-panel organisms; the recovery of each target organism from a blood culture bottle was therefore not verified in a controlled inoculation, although each was shown to be detected from spiked whole blood at a defined inoculum in the analytical sensitivity experiments. Finally, the turnaround times presented here are the durations specified by each workflow’s protocol rather than measured intervals in the clinical cohort: specimen-level laboratory information system timestamps were not collected, so the mean and range of the realised sample-to-result interval could not be determined. The realisable time advantage will in any case depend on local staffing and on whether the platform is operated around the clock, and a prospective evaluation recording actual result-release times is needed before a time saving can be quantified for routine practice.

## 4. Materials and Methods

### 4.1. Study Design and Clinical Specimens

This was a prospective, single-centre, preliminary diagnostic-agreement study conducted in the Division of Clinical Pathology of Tri-Service General Hospital, a 1800-bed tertiary academic medical centre in Taipei, Taiwan. Patients were recruited from the emergency department, the general medical and surgical wards and the intensive care units between January 2025 and January 2026 (enrolment period, 13 months). Eligible patients were adults (≥20 years) in whom the attending physician had ordered blood cultures for clinically suspected bloodstream infection and from whom an additional 5 mL of whole blood could be drawn into a K_2_EDTA tube during the same venepuncture as the blood culture set; patients from whom the additional specimen could not be obtained, and specimens for which any one of the five comparator methods could not be completed, were not enrolled. Sampling was consecutive during the periods in which the study instrument and study staff were available. Forty-three specimens were analysed. No formal sample size calculation was performed: the sample size was determined by the number of specimens that could be processed within the study period, and the clinical component is accordingly reported as an exploratory, preliminary evaluation. Specimens were analysed in de-identified form under the terms of the institutional review board approval, which did not extend to linkage with the electronic medical record; patient-level clinical variables such as the suspected source of infection, prior antimicrobial exposure and severity scores were therefore not available for this analysis. All procedures were conducted following the Declaration of Helsinki and were approved by the Institutional Review Board of Tri-Service General Hospital (TSGHIRB No.: B202405085; approved on 24 May 2024).

### 4.2. Reference Organisms and Reagents

For analytical sensitivity (LoD) evaluation, 10 reference organisms representing the ESKAPE group and additional clinically relevant pathogens were obtained from the American Type Culture Collection (ATCC) or the National Collection of Type Cultures (NCTC) (Table 6).

### 4.3. Nucleic Acid Extraction Methods

Two nucleic acid extraction methods were compared:

- QIAamp DNA Blood Midi Kit (Qiagen^®^, Hilden, Germany): Manual extraction from 2 mL whole blood following the manufacturer’s standard protocol, with elution in 300 μL.
- LabTurbo AIO BPID Mini Kit (LabTurbo Biotech, Taipei, Taiwan): Automated extraction from 5 mL whole blood using the LVRE strategy. Each 1 mL aliquot was processed through a separate mini-column extraction unit, and the pooled nucleic acids from all five columns were concentrated into 50 μL via reverse elution on the LabTurbo SP-qPCR All-in-one system.

### 4.4. Multiplex PCR Detection

Real-time multiplex PCR was performed using the LabTurbo AIO BPID Testing Kit on the LabTurbo SP-qPCR All-in-one system, which integrates nucleic acid extraction, PCR reaction setup via liquid handling, and real-time PCR detection within a single automated platform. The BPID panel comprises pathogen identification targets, which cover both bacterial and fungal species, together with antimicrobial resistance gene targets comprising *mecA*/C, CTX-M, NDM, KPC, OXA-48-like, and *vanA/B*. All testing reported in this study, comprising the index assay, the two comparator molecular assays and all culture-based testing, was performed by the staff of the accredited clinical laboratory of Tri-Service General Hospital. At the time of writing, the LabTurbo AIO BPID panel is supplied by the manufacturer for research use only and is not yet listed as a registered in vitro diagnostic device; it is available on request from LabTurbo Biotech (https://labturbo.com/, accessed on 8 September 2026).

### 4.5. Comparator Methods

Clinical samples were concurrently tested by: (1) conventional blood culture followed by MALDI-TOF MS identification; (2) the BioFire^®^ FilmArray BCID2 panel performed on positive blood culture bottles; and (3) the Qiagen^®^-based direct-from-blood workflow using manual extraction from 2 mL blood followed by the same multiplex PCR detection kit. Blood cultures were collected as part of routine clinical care, and in some patients more than one bottle was collected during the same sampling episode. One set was designated in advance as the study set, and the blood culture column of Supplementary Table S3 reports the result of that designated set only. The FilmArray^®^ BCID2 panel was performed on broth from whichever bottle of the sampling episode flagged positive on the BD BACTEC FX instrument, and MALDI-TOF MS identification was performed on colonies recovered from that bottle on subculture; MALDI-TOF MS was not performed from a bottle that remained culture-negative. A specimen may therefore be recorded as showing no growth in the blood culture column while a concurrently collected clinical bottle from the same episode flagged positive and provided the material for BCID2 and for MALDI-TOF MS identification. Supplementary Table S3 identifies the source bottle for each specimen in which the designated study set and the bottle used for BCID2 and MALDI-TOF MS differ. The BPID^®^ system was applied to the separate K_2_EDTA whole-blood specimen drawn during the same venepuncture and was not exposed to culture broth at any stage.

### 4.6. Quality Control, Contamination Control and Adjudication of Molecular-Only Positive Results

Controls were applied at three levels. (i) Assay-level controls: every BPID^®^ and Qiagen^®^ multiplex PCR run included a no-template control and an internal extraction control added to each specimen before lysis, and a run was reported only when the no-template control was negative and the extraction-control Ct fell within the validated range; nucleic acid extraction, PCR setup and amplification were physically separated, and on the BPID^®^ platform these steps are performed in a closed, single-instrument workflow that minimises amplicon carry-over. (ii) Matrix controls: aliquots of pooled whole blood from healthy donors, confirmed culture-negative, were processed through the complete LVRE workflow in each analytical batch as negative extraction controls. (iii) Blood-culture-level controls: two groups of blood culture bottles were prepared and processed by blood culture, MALDI-TOF MS and the BPID^®^ system, as described in Section 4.7.

### 4.7. Preparation and Testing of Blood Culture Controls

Two groups of blood culture bottles were prepared to verify that the workflow generates no signal in the absence of any organism and does not misassign an organism that lies outside the detection panel. Pooled whole blood from healthy volunteers, confirmed culture-negative by a preceding aerobic and anaerobic set, was used as the matrix. Group B (controls of non-detection, ten bottles) comprised five BD BACTEC Plus Aerobic/F bottles inoculated with 5 mL of the pooled culture-negative blood and no organism and five bottles containing culture medium alone; all were incubated for the full five-day protocol. Group C (off-panel specificity controls, six bottles) used organisms represented on neither the BPID^®^ nor the BCID2 panel, obtained as characterised reference strains from the American Type Culture Collection (ATCC) and held in the departmental strain collection, each spiked into a bottle containing 5 mL of the pooled blood at an inoculum of 100 CFU per bottle. The six were selected as near neighbours of on-panel targets rather than as taxonomically remote organisms, because cross-reactivity is most likely to arise between closely related species: *Acinetobacter lwoffii* ATCC 15309, which lies outside the *Acinetobacter calcoaceticus*–*baumannii* complex covered by the panels; *Enterococcus gallinarum* ATCC 49573, which is distinct from the *Enterococcus faecalis* and *Enterococcus faecium* targets and carries the intrinsic *vanC-1* determinant, so that the *vanA/B* assay of the BPID^®^ panel is also challenged; *Pseudomonas putida* ATCC 12633, a non-*aeruginosa Pseudomonas* species; *Burkholderia cepacia* ATCC 25416, a Gram-negative non-fermenter absent from both panels; *Micrococcus luteus* ATCC 4698, a Gram-positive coccus outside the genus-level *Staphylococcus* target; and *Corynebacterium striatum* ATCC 6940. Two of these correspond directly to organisms encountered in the clinical cohort and to the discordances discussed above, namely the *Acinetobacter* species recovered from Sample 24 and the *Enterococcus* species reported by MALDI-TOF MS in Sample 2, so the control interrogates specific discordances observed in this study. All six grow in aerobic blood culture and are reliably identified by MALDI-TOF MS, so that a failure of the molecular assay to report them can be distinguished from a failure of the organism to be recovered. Species identity was confirmed by MALDI-TOF MS before use. All bottles were loaded into the BD BACTEC FX instrument and monitored under the routine protocol; for Group C an aliquot of the spiked blood taken before incubation was processed in parallel by the BPID^®^ system so that the culture-independent assay was challenged with the same material at the same organism burden as the bottle rather than with an enriched broth. On flagging, or at the end of the incubation period for bottles that did not flag, Gram stain and subculture with MALDI-TOF MS identification were performed.

A separate group of blood culture bottles inoculated with characterised control organisms was not prepared, because the positive-control function for the index test is discharged by the analytical sensitivity experiments described in Section 4.8: each of the ten reference organisms was spiked into culture-negative whole blood at defined inocula and processed through the complete BPID^®^ workflow, in triplicate at five dilution levels for the preliminary determination and in twenty replicates at four concentrations for confirmation, so that every organism on which the assay was evaluated has been shown to be recovered and correctly reported from spiked whole blood at a known concentration. What those experiments do not include is passage through a blood culture bottle; the recovery of each organism from a bottle was therefore not verified in a controlled inoculation, and this is acknowledged in the Limitations.

### 4.8. Analytical Sensitivity (LoD) Protocol

Negative whole blood was spiked with serial dilutions of each reference organism. For possible LoD determination, five 10-fold dilution levels were tested in triplicate; the lowest concentration with a 100% detection rate was designated as the possible LoD. For LoD confirmation, 20 replicates were tested at four concentrations near the possible LoD; the confirmed LoD was defined as the lowest concentration at which at least 95% of replicates were detected, following the general framework of CLSI guideline EP17-A2 for the evaluation of detection capability. An extraction control was included in each replicate to monitor extraction consistency.

### 4.9. Statistical Analysis

PPA, defined as TP/(TP + FN), was calculated at the organism level against a composite reference standard. An organism was classified as reference-positive when identified by at least two of the five methods; organisms identified by a single method only were not counted as reference-positive and were excluded from the denominator. Coagulase-negative staphylococci were matched at the CoNS level and *Proteus* spp. at the genus level. Because two of the five comparators (the BPID^®^ system and the Qiagen^®^ workflow) share the same multiplex PCR detection chemistry and differ only in the extraction step, they were not fully independent. Exact 95% confidence intervals were calculated by the Wilson score method, and paired comparisons between the BPID^®^ system and each comparator used the exact McNemar test. Mean Ct values, standard deviations, and coefficients of variation were calculated for LoD confirmation data. Because the primary composite reference standard incorporated the index test, a post hoc sensitivity analysis requested during peer review was performed in which reference-positive status required identification by at least two of the four non-index methods; PPA and exact McNemar tests were recomputed under this definition and are reported in Section 2.2.3. All discordant organism-level results under either definition are listed individually in Supplementary Table S5. The study was designed as a diagnostic-agreement study; no equivalence or non-inferiority margin was pre-specified, and a non-significant McNemar test is therefore reported as an absence of a detected difference rather than as evidence of equivalence. No adjustment for multiple comparisons was applied, and the *p* values should be interpreted descriptively. Analyses were performed using GraphPad Prism version 9 (GraphPad Software, Boston, MA, USA); confidence intervals for proportions were obtained by the Wilson/Brown hybrid method implemented in that software.

## 5. Conclusions

The automated BPID^®^ system, employing an LVRE nucleic acid extraction strategy, substantially improves PCR-based diagnostic sensitivity for the detection of low-level bacteremia. The LVRE approach addresses the volume–concentration trade-off that has limited existing direct-from-blood molecular methods, achieving confirmed LoD values of 0.67–20.81 CFU/mL, an 8.8- to 24.3-fold improvement over conventional Midi Column extraction. In clinical evaluation, the BPID system demonstrated an organism-level PPA of 95.74% for pathogen identification and 100% concordance for antimicrobial resistance gene detection, higher than the agreement observed for the designated study blood culture set, for MALDI-TOF MS and for the conventional Qiagen^®^ workflow, and yielding organism-level agreement numerically similar to that of a post-culture molecular panel. Because the study was not designed as an equivalence or non-inferiority trial, and because the composite reference standard incorporated the index test, this similarity should not be read as demonstrated equivalence, and the reported agreement should be regarded as an upper bound. With a protocol-specified sample-to-result turnaround time of approximately 3.5 h on the automated platform, this culture-free and fully automated approach has the potential to shorten the diagnostic interval in sepsis and to enable earlier targeted antimicrobial therapy, principally by removing the culture-incubation step rather than by outperforming post-culture molecular panels. These results should be regarded as a preliminary clinical evaluation. Multicentre prospective studies that enrol consecutive patients, include culture-negative and uninfected control specimens, use a clinically adjudicated reference standard that does not incorporate the index test, and measure patient-centred outcomes are warranted before the system is adopted for routine diagnostic use.

## Figures and Tables

**Table 1:** Possible limit of detection (LoD) for QIAamp DNA Blood Midi Kit and LabTurbo AIO BPID Mini Kit extraction methods (CFU/mL).

Target Organism	QIAamp Midi Kit (CFU/mL)	BPID Mini Kit (CFU/mL)	Fold Improvement
*Escherichia coli*	5.2	0.52	10.0×
*Candida tropicalis*	52.2	5.22	10.0×
*Enterococcus faecium*	66	6.6	10.0×
*Acinetobacter baumannii*	86.7	8.67	10.0×
*Klebsiella pneumoniae*	100	10	10.0×
*Enterobacter cloacae*	193	19.3	10.0×
*Pseudomonas aeruginosa*	247	24.7	10.0×
*Serratia marcescens*	247	24.7	10.0×
*Proteus mirabilis*	367	36.7	10.0×
*Staphylococcus aureus*	407	40.7	10.0×

Abbreviations: CFU, colony forming units.

**Table 2:** Confirmed limit of detection (LoD) for both extraction methods, defined as the lowest concentration achieving ≥95% detection across 20 replicates (CFU/mL).

Target Organism	QIAamp Midi Kit (CFU/mL)	BPID Mini Kit (CFU/mL)	Fold Improvement
*Escherichia coli*	6.51	0.67	9.7×
*Klebsiella pneumoniae*	21.16	1.30 ^a^	16.3×
*Enterobacter cloacae*	23.95	2.60	9.2×
*Candida tropicalis*	50.13	5.20	9.6×
*Staphylococcus aureus*	51.65	5.29	9.8×
*Enterococcus faecium*	52.08	2.14 ^a^	24.3×
*Acinetobacter baumannii*	76.67	8.46	9.1×
*Serratia marcescens*	84.00	8.03 ^a^	10.5×
*Proteus mirabilis*	114.38	12.98 ^a^	8.8×
*Pseudomonas aeruginosa*	224.61	20.81	10.8×

Abbreviations: CFU, colony forming units. ^a^ Detection remained ≥95% at the lowest concentration tested; therefore, the LoD was not bracketed and the reported value represents an upper bound.

**Table 3:** Positive percent agreement (PPA) for pathogen identification across five diagnostic methods, calculated at the organism level against a composite reference standard (*n* = 47 reference-positive organisms in 42 samples).

Method	TP	FN	PPA (%)	95% CI	*p* vs. BPID ^b^
BPID^®^ system	45	2	95.74	85.8–98.8	—
FilmArray BCID2	44	3	93.62	82.8–97.8	1.00
Qiagen^®^ method	34	13	72.34	58.2–83.1	0.001
Blood culture	34	13	72.34	58.2–83.1	0.007
MALDI-TOF MS	35	12	74.47	60.5–84.8	0.013

Abbreviations: TP, true positive (a reference-positive organism that was detected by the method); FN, false negative (a reference-positive organism that was not detected by the method); PPA, positive percent agreement; CI, confidence interval. ^b^ Exact McNemar test comparing each method with the BPID^®^ system on the 47 reference-positive organisms.

**Table 4:** Specimen-level concordance with the BCID2 reference for antimicrobial resistance determinants covered by both panels (*n* = 16 specimens in which the BCID2 reference reported at least one such determinant).

Method	TP	FN	PPA (%)
BPID^®^ system	16	0	100
Qiagen^®^ method	10	6	62.5

The unit of analysis is the specimen, not the individual determinant. Abbreviations: TP, true positive (a specimen in which the method reported the same set of shared-coverage determinants as the BCID2 reference); FN, false negative (a specimen in which the method did not reproduce that determinant profile); PPA, positive percent agreement. Determinants covered by only one of the two panels, including IMP and VIM, lie outside the shared coverage and were not analysed. MRSA and VRE are phenotypic designations used in Supplementary Table S3 to denote detection of *mecA/C* and of *vanA/B*, respectively.

**Table 5:** Results of the blood culture control experiments.

Control Group and Organism	Inoculum (CFU/Bottle)	Blood Culture	MALDI-TOF MS (Identification)	BPID^®^ System (Organism, AMR Genes)
Group B—controls of non-detection (*n* = 10 bottles)				
Culture-negative pooled whole blood, no organism (*n* = 5)	None	No growth	Not applicable	Not detected
Culture medium alone, uninoculated (*n* = 5)	None	No growth	Not applicable	Not detected
Group C—off-panel specificity controls (*n* = 6 bottles)				
*Acinetobacter lwoffii* ATCC 15309	100	Positive	*Acinetobacter lwoffii*	Not detected
*Enterococcus gallinarum* ATCC 49573 (vanC-1)	100	Positive	*Enterococcus gallinarum*	Not detected; no *vanA/B* signal
*Pseudomonas putida* ATCC 12633	100	Positive	*Pseudomonas putida*	Not detected
*Burkholderia cepacia* ATCC 25416	100	Positive	*Burkholderia cepacia*	Not detected
*Micrococcus luteus* ATCC 4698	100	Positive	*Micrococcus luteus*	Not detected
*Corynebacterium striatum* ATCC 6940	100	Positive	*Corynebacterium striatum*	Not detected

For Group C the BPID^®^ system was applied to an aliquot of the spiked blood taken before incubation, so that the culture-independent assay was challenged with the same material at the same organism burden as the bottle. The FilmArray BCID2 panel and the Qiagen^®^ workflow were not repeated on control material: BCID2 is a commercially validated panel whose analytical specificity is established by the manufacturer, and the Qiagen^®^ workflow uses the identical multiplex PCR detection kit as the BPID^®^ system and differs only in the extraction step, so the specificity result obtained for the BPID^®^ system applies equally to it. Expected results: no growth and no detection (Group B); recovery by culture and MALDI-TOF MS with no report by the BPID^®^ system and no cross-reactive on-panel identification (Group C). The Group C organisms were chosen as near neighbours of on-panel targets, since cross-reactivity is most likely between closely related species; the *vanC*-carrying enterococcus additionally tests whether the *vanA/B* assay cross-reacts with *vanC*. The threshold applied for species-level MALDI-TOF MS assignment in our accredited workflow is a score of ≥2.0.

**Table 6:** Reference organisms used for LoD evaluation.

Target Organism	Source	Catalog No.	Lot No.	Conc. (CFU/mL)
*Enterococcus faecium*	ATCC 700221	01000L	1000-31	9.9 × 10^7^
*Staphylococcus aureus*	ATCC 29213	0365L	365-113	6.1 × 10^8^
*Klebsiella pneumoniae*	ATCC BAA-1705	01005L	1005-37	1.5 × 10^8^
*Acinetobacter baumannii*	NCTC 13304	01266L	1266-05	1.3 × 10^8^
*Pseudomonas aeruginosa*	ATCC 27853	0353L	353-479	3.7 × 10^8^
*Enterobacter cloacae*	NCTC 13464	01105L	1105-11	2.9 × 10^8^
*Escherichia coli*	ATCC 25922	0335L	335-538	7.8 × 10^6^
*Serratia marcescens*	ATCC 13880	0247L	247-51	3.7 × 10^8^
*Proteus mirabilis*	ATCC 35659	0944L	944-74	5.5 × 10^6^
*Candida tropicalis*	ATCC 750	0847L	847-48	1.8 × 10^6^

## Data Availability

The datasets generated and analyzed in this study are not publicly available due to legal and ethical restrictions imposed by the Tri-Service General Hospital Institutional Review Board (TSGHIRB No. B202405085). De-identified data can be made available from the corresponding author upon reasonable request and with prior approval from the TSGHIRB.

## Supplementary Materials

The following supporting information can be downloaded at https://www.mdpi.com/article/10.3390/antibiotics15090896/s1: Table S1: Complete LoD confirmation raw Ct data for QIAamp DNA Blood Midi Kit; Table S2: Complete LoD confirmation raw Ct data for LabTurbo AIO BPID Mini Kit; Table S3: Clinical evaluation—complete pathogen identification results for all 43 samples across five methods; Table S4: Per-case composite reference adjudication for the 47 reference-positive organisms; Table S5: Complete listing of all discordant organism-level results across the five methods, including the sample identifier, the organisms reported by each method, the classification under the primary and the index-test-excluded reference standards, and the classification of molecular-only detections; Table S6: Results of the sensitivity analysis using a composite reference standard that excludes the index test; Figure S1: Nucleic acid concentration and A260/280 and A260/230 purity ratios for the 18 paired specimens processed by LVRE and Midi kit extraction (revised for clarity).

## Abbreviations

The following abbreviations are used in this manuscript:

LVRE	large-volume reverse-elution
LoD	limit of detection
CFU	colony forming unit
PPA	positive percent agreement
TP	true positive
FN	false negative
BSI	bloodstream infection
AMR	antimicrobial resistance
AMS	antimicrobial stewardship
NPA	negative percent agreement
CI	confidence interval
CoNS	coagulase-negative staphylococci
TAT	turnaround time
BCID2	BioFire FilmArray Blood Culture Identification 2 panel
MALDI-TOF MS	matrix-assisted laser desorption/ionization time-of-flight mass spectrometry
NTC	no-template control
IQR	interquartile range
