# Supplementary material for: Analytical and Clinical Evaluation of a Fully Automated Direct-from-Whole-Blood Multiplex PCR Assay Using Large-Volume Reverse Elution for Rapid Bloodstream Infection Diagnosis
Source: Antibiotics (Basel). 2026 Sep 11;15(9):896. doi: 10.3390/antibiotics15090896 (PMC13603350; doi:10.3390/antibiotics15090896)
Supplement: Supplementary file 1 [file antibiotics-15-00896-s001.zip › Figure_S1_2026-09-11.pdf]

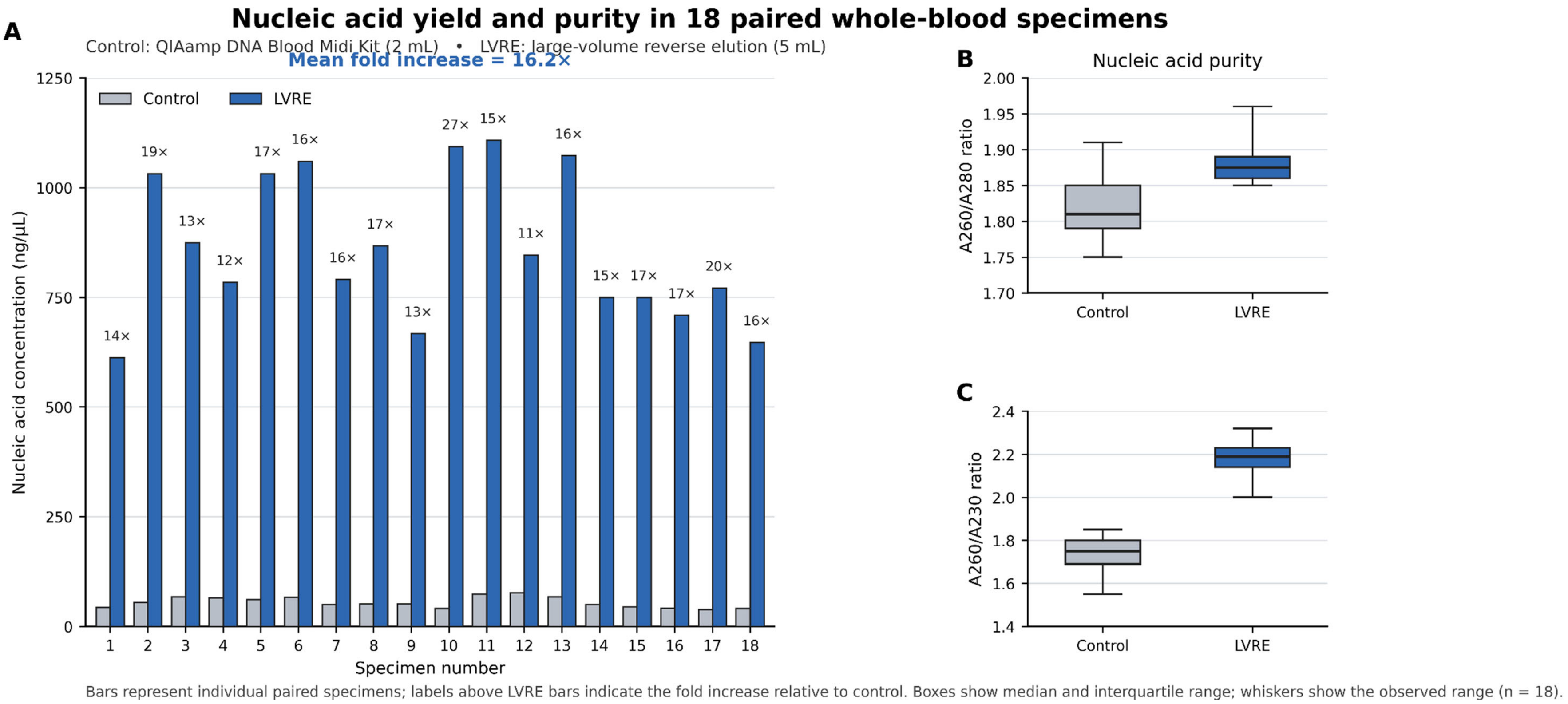


**Figure S1.** Nucleic acid yield and purity in 18 paired whole-blood specimens. Each specimen was processed in parallel by the QIAamp DNA Blood Midi Kit control (2 mL input) and by the large-volume reverse-elution (LVRE) workflow (5 mL input). (A) Nucleic acid concentration (ng/µL) for each of the 18 paired specimens; the label above each LVRE bar is the fold increase relative to its paired control, and the mean fold increase was 16.2×. (B) A260/A280 ratio and (C) A260/A230 ratio, both dimensionless; boxes show the median and interquartile range and whiskers the observed range ($n = 18$ per group).
